# Synergistic effect of naphthalene acetic acid and salicylic acid on the growth and tolerance mechanism of cucumber under salt stress

**DOI:** 10.1038/s41598-026-39439-x

**Published:** 2026-03-14

**Authors:** Esraa Mohamed, Sayed Hussein Abdelgalil, Mohamed Omar Kaseb, Islam I. Teiba, Sobhi F. Lamlom, Ahmed M. Abdelghany, Diaa Abd El-Moneim, Mohamed E. Shalaby

**Affiliations:** 1 Cross Pollination Vegetables Dep., Horticulture Research Institute, Agricultural Research Center (ARC), Dokki, Giza, Egypt; 2 Central Laboratory of Organic Agriculture (CLOA), Agricultural Research Center (ARC), Giza, Egypt; 3 Botany Department, Faculty of Agriculture, Tanta University, Tanta City, 31527 Egypt; 4 Plant Production Department, Faculty of Agriculture Saba Basha, Alexandria University, Alexandria, 21531 Egypt; 5 Crop Science Department, Faculty of Agriculture, Damanhur University, Damanhur, 22516 Egypt; 6 Department of Plant Production, (Genetic Branch), Faculty of Environmental and Agricultural Sciences, Arish University, El-Arish, Egypt

**Keywords:** Abiotic stress mitigation, Antioxidant enzymes, Metabolic regulation, Oxidative stress response, Crop resilience, Salt tolerance, Biochemistry, Physiology, Plant sciences

## Abstract

High soil salinity significantly hampers plant growth and crop yield by disrupting metabolic functions and causing oxidative, osmotic, and ionic stress. This study investigated the combined effects of naphthalene acetic acid (NAA) and salicylic acid (SA) in reducing salt stress in cucumber (*Cucumis sativus* L.) cv. AR-8, filling a knowledge gap regarding multilateral plant growth regulator (PGR) interactions under saline conditions. Cucumber seedlings were subjected to 80 mM NaCl, with or without 200 ppm NAA and 300 ppm SA. Salt stress notably decreased seedling vigor, biomass, and chlorophyll levels. However, combined NAA and SA treatments significantly mitigated these effects, leading to improvements in leaf area, plant height, and root activity compared to untreated stressed plants. The PGRs also enhanced fruit quality by increasing vitamin C, soluble sugars, and protein content. Additionally, they reduced oxidative stress markers such as electrolyte leakage and lipid peroxidation, while boosting antioxidant enzyme activities like catalase, ascorbate peroxidase, superoxide dismutase, and peroxidase. The treatments further influenced carbohydrate and nitrogen metabolism by modulating key enzymes, including sucrose phosphate synthase, glutamine synthetase, glutamate synthase, and glutamate dehydrogenase. This research highlights the synergistic effect of NAA and SA in promoting cucumber tolerance to salt stress and offers new insights into using multiple PGRs for crop resilience in saline environments. Future studies should aim to optimize PGR combinations and explore their molecular mechanisms in different crops under various stress conditions.

**Supplementary Information:**

The online version contains supplementary material available at 10.1038/s41598-026-39439-x.

## Introduction

Abiotic stress presents a substantial obstacle to worldwide agricultural output, impacting plant development and crop yields^1–3^. Soil salinity is a significant abiotic stress, exacerbated by global climate change and unsustainable activities, including inadequate irrigation^1,4^. Salt stress negatively affects plant growth and development, especially in crops such as cucumber (*Cucumis sativus* L.) and other members of the Cucurbitaceae family, which exhibit considerable sensitivity to salty environments^5,6^. Such stress adversely affects metabolic processes, impairing nitrogen absorption, diminishing reduction, and compromising protein synthesis, ultimately leading to decreased growth vigor and overall yield^7^. Elevated salt concentrations affect the intracellular equilibrium of carbon and nitrogen, hinder metabolic processes, and impede chlorophyll synthesis, hence exacerbating growth inhibition^8^. Furthermore, salt stress induces osmotic stress by diminishing soil osmotic potential, impairing water absorption, and net photosynthetic yield^9^. The buildup of reactive oxygen species (ROS), including superoxide anions (O₂⁻•), within the electron transport pathways of chloroplasts, mitochondria, and peroxisomes leads to oxidative stress, resulting in membrane impairment and the disruption of essential processes such as respiration and photosynthesis^10^. The susceptibility of cucumbers and similar crops to salt stress requires investigating efficient mitigation measures to maintain productivity in saline conditions^11^.

Plant growth regulators (PGRs) have been demonstrated to modulate essential physiological processes in plants, encompassing growth, development, and reactions to abiotic stressors^12^. Naphthalene acetic acid (NAA) and salicylic acid (SA) are useful in improving plant resilience under salt stress. Plant growth regulators, whether naturally occurring or synthetic, function as chemical messengers that affect multiple facets of plant growth, including cell division, elongation, and stress resilience^13^. Numerous recent studies have underscored the advantages of integrating NAA and SA to enhance overall plant growth, photosynthesis, and stress resilience in saline environments^12,14^. The positive effects of NAA on plant growth, especially in improving yield and photosynthesis in the face of different stress situations, are becoming more and more well-known^15^. There is strong evidence from recent studies that NAA is effective in improving plant health and resilience generally. Results showed that when applied to avocado trees at 30 ppm NAA concentrations, there was a marked improvement in fruit set and a decrease in fruit drop when compared to the control treatments^16^. Across all seasons, NAA boosted fruit yield metrics such as kg/tree and ton/feddan by stimulating cell elongation and enhancing vacuole enlargement^16^. Studies have demonstrated that NAA supports photosynthesis by enhancing chlorophyll synthesis and improving photosynthetic efficiency. This function is especially critical for maintaining crop productivity under abiotic stresses, such as salinity^17^. Sustainable agricultural development can be advanced through the incorporation of NAA into farming techniques, which has the potential to make crops more resistant to environmental stresses^18^.

SA is essential for regulating vital physiological processes that augment stress tolerance, specifically by enhancing photosynthetic efficiency and chlorophyll production under unfavorable environmental conditions, where ionic imbalances may disrupt cellular functions^19^. The foliar application of SA has demonstrated enhanced plant growth and physiological responses under stress conditions, including heavy metal exposure and salt stress. In barley, the application of SA at concentrations of 0.5 to 1 mg L⁻¹ markedly enhanced plant height, biomass, chlorophyll content, and photosynthetic rates, thereby alleviating the detrimental effects of salinity^20^. Lettuce treated with SA (100–500 mg L⁻¹) demonstrated improved plant height and root length, decreased cadmium absorption, and elevated antioxidant enzyme activity, hence protecting against cadmium-induced oxidative stress^21^. Furthermore, the application of SA in maize enhanced the synthesis of phenolic compounds and flavonoids, critical for plant defense, and increased antioxidant enzyme activity, thereby bolstering the plant’s resistance to both biotic and abiotic stressors^22^.

Although research on the combined effects of SA and NAA is limited, studies suggest their potential to enhance crop growth and photosynthetic efficiency. For example, foliar application of SA and NAA in mungbean (*Vigna radiata*) significantly increased plant height, biomass, and yield, with NAA at 80 ppm showing the greatest improvements^23^. Despite these findings, no research has thoroughly examined how these two PGRs affect defense mechanisms under salt stress, particularly in cucumber (Cucumis sativus). Such NAA-SA crosstalk involves multiple molecular mechanisms, including the modulation of auxin transporters by SA and the regulation of defense-related genes by auxin signaling components^24^. This interaction is particularly relevant as many crops face various environmental stresses during their growth cycle, affecting both yield and quality^24^. The SA signaling pathway has been shown to interact with auxin-mediated growth responses through the regulation of transport inhibitor response 1 (TIR1) and various auxin response factors (ARFs), suggesting a complex regulatory network that balances growth and defense responses^24^. NAA, as a synthetic auxin, regulates metabolism through several distinct pathways. It promotes cell elongation via the acid growth hypothesis, where auxins stimulate H⁺-ATPase activity in the plasma membrane, leading to cell wall loosening and subsequent elongation. Additionally, NAA influences gene expression through the TIR1/AFB-Aux/IAA-ARF signaling pathway. When NAA binds to the TIR1/AFB receptor complex, it enhances the degradation of Aux/IAA repressor proteins, thereby freeing ARF transcription factors to modulate gene expression^25^. Under salt stress conditions, NAA has been shown to upregulate genes involved in ROS scavenging, especially those encoding superoxide dismutase and catalase^26^. Furthermore, NAA enhances nutrient uptake by regulating ion transporter expression in root cells, particularly K⁺ transporters, which help maintain favorable K⁺/Na⁺ ratios during salt stress^15^.

Cucumber (*Cucumis sativus* L.) was selected for this study due to its global significance as one of the most widely cultivated and economically important vegetable crops^27^. It is highly sensitive to abiotic stresses such as salinity, which substantially threatens its productivity^28^. Cucumber is crucial in human nutrition, providing essential vitamins, minerals, and antioxidants^29^. Additionally, its fast growth cycle and relative ease of cultivation make it an ideal model species for studying the effects of environmental stressors on plant physiology^30^. Economically, cucumber is a high-value crop, contributing significantly to the agricultural sectors of many countries. In regions where salinity is an increasing concern, adjust the expression “2:1 ratio, peat moss and sand”. Understanding strategies to mitigate its adverse effects on cucumber production is essential for maintaining both yield and quality, thereby ensuring the crop’s sustainability and profitability in salt-affected areas^31^. The originality of this study resides in addressing a critical gap by investigating the synergistic impacts of Salicylic Acid (SA) and Naphthalene Acetic Acid (NAA) on antioxidant defense, nutrient uptake, carbohydrate and nitrogen metabolism in saline conditions. This research represents the inaugural examination of whether the concomitant application of SA and NAA constitutes a more efficacious strategy for alleviating salt-induced damage and enhancing cucumber resilience. Consequently, the primary objective of this study was to assess the effects of SA, NAA, and their interaction on plant growth, physiological responses, biochemical adaptations, and fruit quality in the context of 80 mM NaCl-induced salinity.

## Materials and methods

### Seed germination and seedling establishment

Seeds of cucumber of the cultivar AR-8 were obtained from the Agriculture Research Center, Ministry of Agriculture, Giza, Egypt. Seeds were placed in Petri dishes (9*9 cm) on filter paper moistened by sterile water and incubated for approximately 24 h at 28 ± 10 °C. After incubation, germinated seeds were cultivated in the trays until they had 2 true leaves (21 days). Seedlings were maintained under controlled conditions with a 16/8-hour (light/dark) photoperiod and light intensity of 450 µmol m⁻² s⁻¹ PPFD. The plantlets were then transferred to plastic containers (50*70 cm) placed in the greenhouse after being filled with a cultivation substrate of peat moss and sand at a 2:1 ratio (v/v), with pH 6.2. The substrate was pre-fertilized with NPK (14:10:14) slow-release fertilizer. NaCl, NAA (naphthalene Acetic Acid 98% Powder, ITACO. CO. Let. Egypt), and SA (salicylic Acid 99% Powder, ALFA Chem. Egypt) were thoroughly mixed with the cultivation substrate outside of the containers, and then seedlings were transplanted into these containers on the same day. The experiment was conducted during the summer season (April to July), which has a hot, dry climate. Temperature in the greenhouse was maintained at 28 ± 10 °C during the day and 18 ± 2 °C at night, with relative humidity of 65 ± 5%.

### Salt stress experiment

This study was executed in a completely randomized design (CRD) with three replications and five pots for each treatment. A random sample was selected to determine the parameters. The experiment was conducted with three treatments: S1—control; S2- 80 mM NaCl (electrical conductivity 7.4 mS/cm); and S3- 200 ppm NAA + 300 ppm SA + 80 mM NaCl. Irrigation was maintained at 70–75% of field capacity throughout the experiment, monitored using soil moisture sensors. Plants were grown to maturity to evaluate vegetative growth parameters and reproductive development, including fruit production.

### Data recorded

The study consisted of two complementary analyses: (1) evaluation of vegetative growth, physiological responses, and biochemical adaptations in cucumber seedlings under salt stress, and (2) assessment of fruit quality parameters from mature plants. Seedling measurements were conducted on 21-day-old plants, while fruits were harvested from 65-day-old plants during the peak production period to evaluate quality parameters such as vitamin C, soluble sugar, and protein. This comprehensive approach allowed us to assess the immediate physiological responses to salt stress and the subsequent effects on fruit quality and yield.”

#### Physiological parameters

Multiple physiological indicators were assessed to evaluate the impact of different treatments on cucumber seedlings. Measurements were taken during the seedling stage, after 21 days, specifically when the first true leaves were fully expanded, as described in^32^.

##### Seedling vigor index

The seedling vigor index was determined by multiplying the sum of shoot length and root length by the germination % and then multiplying it again by the germination percentage.

$$\:\boldsymbol{V}\boldsymbol{I}=\left(\left(SL+RL\right)\mathrm{*}G\mathrm{\%}\right)$$

where SL is shoot length (cm), RL is root length (cm), and G% is the germination percentage. This index was used to assess the overall vigor of the seedlings, specifically the magnetic field treatment before sowing^33^.

##### Chlorophyll content

The chlorophyll content was quantified using a SPAD-502 chlorophyll meter. The chlorophyll reading measured in SPAD values was recorded from completely expanded leaves to evaluate the photosynthetic capacity under varying durations of red and blue LED illumination^34^.

##### Root and shoot fresh weights

After harvesting, the shoot height and root length were measured using a measuring tape. Next, fresh and dry weights, and shoot and root dry weights of the seedlings were measured using an electric balance. For dry weight determination, the plants were oven-dried at 105° C for 30 min and then dried at 75° C until constant weight^35^. Leaf area was measured with the LI-3100 C leaf area meter by scanning fully expanded leaves, while plant height was measured from the stem base to the main shoot tip using a ruler^35^.

#### Physiological parameters

##### Vitamin C and soluble sugar

The vitamin C content was determined using ascorbic acid (0.01 mg/mL) as the reference compound. Two hundred milliliters of the extract was mixed with 300 mL of 13.3% trichloroacetic acid (TCA) and 75 mL 2,4-dinitrophenylhydrazine (DNPH). The mixture was incubated at 37 °C for 3 h, and 500 mL of H_2_SO_4_ was added to the mixture before the absorbance was read at 520 nm^36^. Total soluble sugars, sucrose, and starch were measured following Buysse and Merckx’s method. For sugar extraction, 200 mg samples were processed five times with 8 mL of 80% ethanol solution, centrifuged, and diluted to 50 mL in volumetric flasks. Total soluble sugars were determined spectrophotometrically using 0.1 mL of the extract, while sucrose determination required 0.4 mL. The centrifugation pellets were dried at 60 °C for 48 h. To measure starch, these dried pellets underwent hydrolysis for 3 h in 10 mL of 2% HCl at 100 °C, followed by centrifugation and volume adjustment to 25 mL. The resulting sugars were measured spectrophotometrically at 620 nm. Standard curves were created using sucrose for the sucrose measurements and glucose for both total sugar and starch determinations.

##### Protein and nitrogen content

Protein and Nitrogen content were determined according to Bremner et al.^37^. After 21 days, tissue samples of leaves and roots were collected during the harvest. These samples were then subjected to oven-drying at a temperature of 68 °C for 48 h. Subsequently, the dried samples were ground to a fine consistency, capable of passing through a 1-mm mesh screen. The Kjeldahl technique was employed to ascertain the total nitrogen content^38^.

#### Physiological stress indicators

##### Electrolyte leakage

Electrolyte leakage (EL) was measured by first collecting five leaf discs (each 1.54 cm² in area) using a copper hole puncher. After washing, these discs were placed in an Erlenmeyer flask containing 50 mL of distilled water. The flask was covered with aluminum foil and maintained at 25 °C for 24 h. Following this incubation period, the solution’s initial electrical conductivity (Xi) was recorded using a benchtop conductivity meter (MB11, MS Techonopon, Piracicaba—SP, Brazil). Subsequently, the flask was heated in an oven (SL100/336, SOLAB) at 80 °C for 120 min. Once cooled, the final electrical conductivity (Xf) was measured. The percentage of electrolyte leakage was then calculated using the equation: % EL = (Xi/Xf) × 100, where Xi represents the initial electrical conductivity and Xf represents the final electrical conductivity, as recommended by reference [34]. distilled water (Milli-Q50, Millipore, Bedford, MA). To facilitate EL from injured tissues at an ambient temperature, a flask was shaken for 20 h. The EC was determined by registering each vial using an Acro met AR20 EC meter (Fisher Scientific, Chicago, IL). The flasks were immersed in a hot water bath (Fisher Isotemp, Indiana, PA) at a temperature of 80 °C (176 °F) for 1 h in order to cause the cell to rupture. The vials were once again positioned on the Innova 2100 platform shaker for a duration of 20 h at a temperature of 21 °C (70 °F). Final conductivity had been recorded per flask. EL% per baud was recorded as the first conductivity/end conductivity × 100.

##### Proline content

Proline extraction (0.2 g samples) was conducted using two ml of 10% acetic acid and five ml of 3% salicylic acid, respectively. The mixture was centrifuged at 12,000 × g for 10 min. The resulting supernatants were analyzed using a standard method. The determination of proline was conducted using the methodology outlined by Aebi^39^.

#### Antioxidant enzymes assay

##### Catalase, peroxidase, and ascorbate peroxidase

To assess the activity of antioxidant enzymes, 0.5 g of freshly harvested leaves from each treatment were finely crushed using a pre-cooled pestle and mortar in 5 mL of phosphate buffer (50 mM, pH 7.8). The uniform mixture was centrifuged at a force of 15,000 times the acceleration due to gravity at a temperature of 4 °C for 15 min. Next, the liquid portion (supernatant) was gathered and utilized to assess the antioxidant capabilities, specifically catalase (CAT), peroxidase (POD), and ascorbate peroxidase (APX). The CAT enzyme activity was evaluated using the Aebi technique^40^ as described in^32^. The reaction mixture consisted of 3 mL volume, containing 100 µL of enzyme extract, 2.8 mL of phosphate buffer with EDTA (2 mM, pH 7.0), and 100 µL of 300 mM H2O2. The CAT activity was assessed by measuring the reduction in absorbance at 240 nm resulting from the breakdown of H_2_O_2_. The reaction mixture comprised 20 mg of total protein, 50 mM of sodium phosphate buffer (pH 7.0), and 10 mM of H2O2. Catalase activity was quantified as a 0.01 reduction in absorbance at 240 nm per mg of protein per minute. The CAT activity was determined using the provided formula: The activity of CAT per unit of fresh weight (Units/g FW) can be calculated using the formula:

$$\:\mathrm{C}\mathrm{A}\mathrm{T}\:\mathrm{a}\mathrm{c}\mathrm{t}\mathrm{i}\mathrm{v}\mathrm{i}\mathrm{t}\mathrm{y}\:=\frac{\mathrm{A}\mathrm{c}\mathrm{t}\mathrm{i}\mathrm{v}\mathrm{i}\mathrm{t}\mathrm{y}\mathrm{*}\mathrm{A}\mathrm{*}\left(\frac{\mathrm{V}}{\mathrm{a}}\right)}{\mathrm{E}\mathrm{*}\mathrm{W}}$$

where: Activity = change in absorbance per minute (ΔA min⁻¹), A = total volume of enzyme extract (mL), V = total reaction volume (mL), a = volume of enzyme extract used in the assay (mL), E = extinction coefficient of H₂O₂ (39.4 mM⁻¹ cm⁻¹ at 240 nm), W = fresh weight of the plant sample (g).

The POD activity was measured by following the protocol as suggested by Pütter^41^. The reaction mixture consisted of 3 mL, containing enzyme extract (0.1 mL), 300 mM H_2_O_2_ (0.1 mL), 1.5% guaiacol (0.1 mL), and 50 mM phosphate buffer (2.7 mL, pH 7.8). The assessment of the POD activity involved spectrophotometric measurement of the increase in absorbance at 470 nm, which was caused by the oxidation of guaiacol. The calculation of the POD activity was determined using the provided formula:

The activity of POD per unit of gram of fresh weight (Units/g FW) is calculated using the formula:

$$\:\mathrm{P}\mathrm{O}\mathrm{D}\:\mathrm{a}\mathrm{c}\mathrm{t}\mathrm{i}\mathrm{v}\mathrm{i}\mathrm{t}\mathrm{y}\:=\frac{\mathrm{A}\mathrm{c}\mathrm{t}\mathrm{i}\mathrm{v}\mathrm{i}\mathrm{t}\mathrm{y}\mathrm{*}\mathrm{A}\mathrm{*}\left(\frac{\mathrm{V}}{\mathrm{a}}\right)}{\mathrm{E}\mathrm{*}\mathrm{W}}$$

where: Activity = change in absorbance per minute (ΔA min⁻¹), A = total volume of enzyme extract (mL), V = total reaction volume (mL), a = volume of enzyme extract used in the assay (mL), E = extinction coefficient of the reaction substrate (e.g., 26.6 mM⁻¹ cm⁻¹ for guaiacol at 470 nm), W = fresh weight of the plant sample (g).

The APX enzyme activity was measured by following the method described by Nakano and Asada^42^. The reaction mixture consisted of 0.1 mL of enzyme extract, 0.1 mL of 7.5 mM ascorbate, 0.1 mL of 300 mM H2O2, and 2.7 mL of 50 mM phosphate buffer with 2 mM EDTA at pH 7.8. The APX oxidation was tracked by measuring the change in absorbance at a wavelength of 290 nm. The APX activity was determined using the provided formula: The APX activity per gram of fresh weight (Units/g FW) is calculated using the formula: Activity multiplied by A multiplied by V divided by a, and then divided by E multiplied by W, as follows:

$\:\mathrm{A}\mathrm{P}\mathrm{X}\:\mathrm{a}\mathrm{c}\mathrm{t}\mathrm{i}\mathrm{v}\mathrm{i}\mathrm{t}\mathrm{y}\:=\frac{\mathrm{A}\mathrm{c}\mathrm{t}\mathrm{i}\mathrm{v}\mathrm{i}\mathrm{t}\mathrm{y}\mathrm{*}\mathrm{A}\mathrm{*}\left(\frac{\mathrm{V}}{\mathrm{a}}\right)}{\mathrm{E}\mathrm{*}\mathrm{W}}$where: Activity = change in absorbance per minute (ΔA min⁻¹), A = total volume of enzyme extract (mL), V = total reaction volume (mL), a = volume of enzyme extract used in the assay (mL), E = extinction coefficient of ascorbate (2.8 mM⁻¹ cm⁻¹ at 290 nm), W = fresh weight of the plant sample (g).

##### Malondialdehyde (MDA)

Heath and Packer^43^, utilized the thiobarbituric acid (TBA) reaction technique, with some adjustments, to measure the MDA contents. A 0.25 g sample of fresh leaves was combined with 5 mL of trichloroacetic acid solution containing 0.1% TCA. Next, the combination underwent centrifugation for 15 min at a force of 12,000 times the acceleration due to gravity (12,000× g). A 1 mL sample was combined with 4 mL of TCA (20%) and TBA (0.5%) in a container. The mixture was then subjected to heating at a temperature of 95 °C for 30 min. Subsequently, it was rapidly chilled in an ice bath and centrifuged for 10 min at a force of 10,000 times the acceleration due to gravity (10,000× g). The MDA contents were computed utilizing the provided formula: The MDA (Units/g FW) can be calculated using the formula:

$$MDA = 6.45*\left( {\left( {A_{{532}} - A_{{600}} } \right) - \left( {0.56*A_{{450}} } \right)} \right)$$

where: A_532_, A_600_, and A_450_ are the absorbance values measured at 532, 600, and 450 nm, respectively; 6.45 is the extinction coefficient used for MDA calculation, FW represents the fresh weight of the sample (g).

#### Key metabolic enzyme activities

The levels of glutamine synthetase (GOGAT), glutamate synthase GS, glutamate dehydrogenase (GDH), sucrose synthase (SPS), and sucrose phosphate synthase (SS) were determined using the procedures described by Hu et al.^39^. For determination of SS and SPS activities, leaves were homogenized with 0.1 M phosphoric acid buffer (pH 7.5), 5 mM MgCl2, 1 mM EDTA, 0.1% mercaptoethanol, and 0.1% Triton X-100 mixture in an ice bath, followed by centrifugation at 12,000×g for 15 min to collect the supernatant. The enzyme activities of SS and SPS were measured using enzyme kits (Cominbio, Suzhou, China) according to the manufacturer’s instructions. The activities of SS were expressed as micrograms (product reducing sugar) per minute per gram77. One SPS activity unit was defined as producing 1 µg sucrose per minute per gram of fresh leaves. GS activity was analyzed using a GS detection kit, and an enzyme activity unit was calculated to produce 1 µmol γ-glutamylhydroxamic acid per hour per gram of fresh leaves per millilitre of the reaction system at 540 nm79. GOGAT and GDH activities were monitored using the absorbance at 340 nm, and the consumption of 1 nmol of NADH per minute per gram tissue was defined as one unit of enzyme activity.

### Statistical analysis

Data was analyzed using one-way ANOVA with repeated measures, followed by Tukey’s post hoc test (p < 0.05) for pairwise comparisons. Statistical analyses and data visualizations were conducted in RStudio (R Core Team, 2024, version 4.3.1). Bar charts were generated with the ‘ggplot2’ package^44^, while Principal Component Analysis (PCA) visualizations utilized the ‘factoextra’^45^ and ‘FactoMineR’ packages^46^. Hierarchical clustering and heatmap visualizations were created using the ‘pheatmap’^47^ and ‘corrplot’ packages^48^. All statistical tests were carried out with the ‘stats’ package (R Core Team, 2024).

## Results

### Effects of salt and plant growth regulators on seedling vigor, chlorophyll content, and plant biomass in cucumber under salt stress

**Fig. 1:**
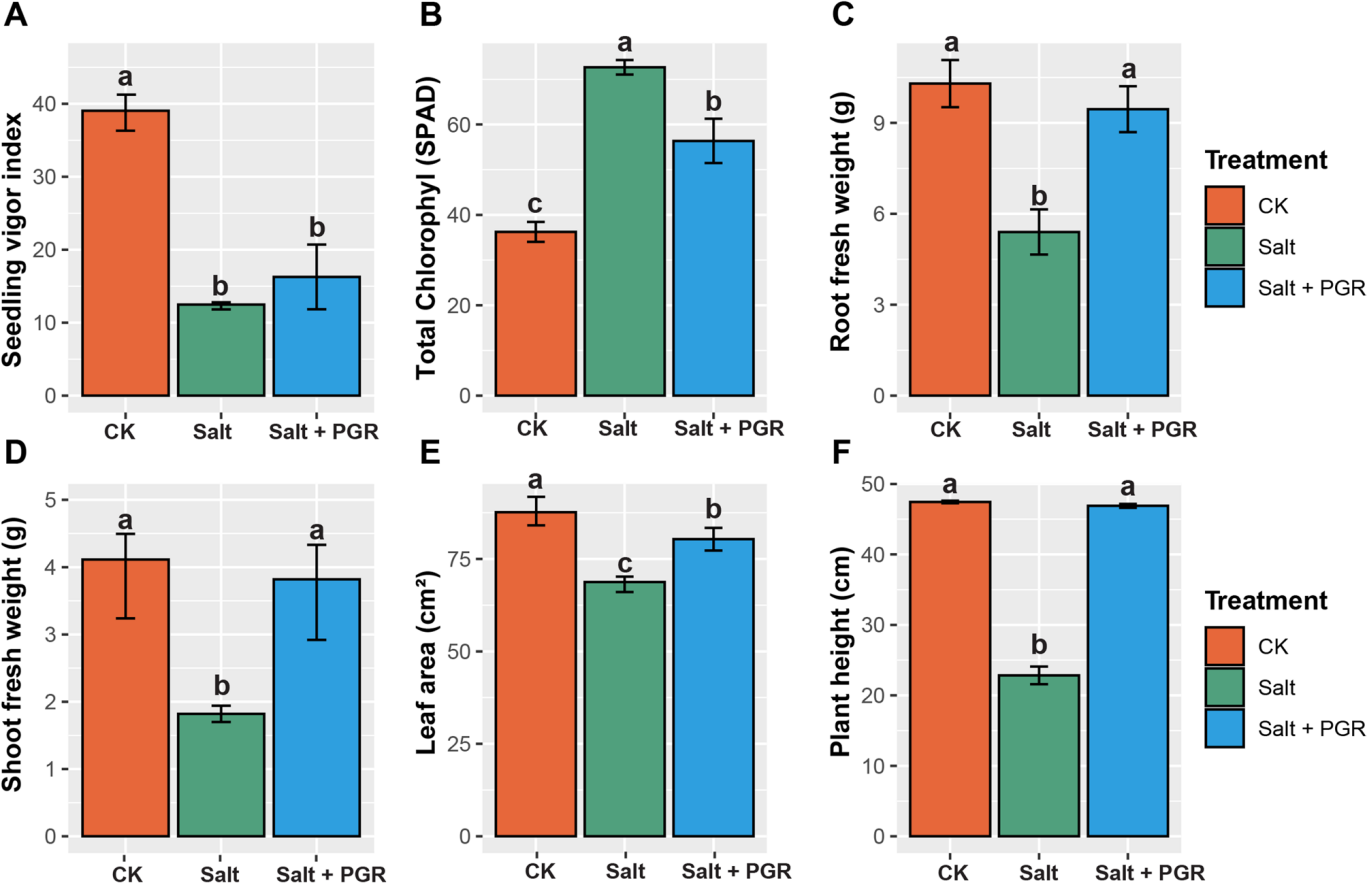
Effect of NAA and SA in various combinations on seedling vigor index (**A**), chlorophyll content (**B**), root fresh weight (**C**), shoot fresh weight (**D**), leaf area (**E**), and plant height (**F**) of cucumber seedlings under 80 mM NaCl stress. CK represents the control group, Salt represents salt stress, and Salt + PGR represents salt stress with a PGR combination (NAA + SA). Distinct letters above the bars indicate significant differences between treatments at a significance level of *p* < 0.05, as determined by Tukey’s HSD test. The data shown represents the average values with standard errors (SE).

The findings presented in (Fig. 1A,F) illustrate the impacts of distinct treatments (CK: control, Salt: salt stress, Salt + PGR: combination of salt stress and plant growth regulators) on a range of cucumber plant parameters. These parameters comprise seedling vigor index, chlorophyll content, root and shoot fresh weight, leaf area, and plant height. Regarding the vigor of seedlings, salt stress significantly reduced seedling vigor by 66.7% compared to the control. However, the Salt + PGR treatment partially alleviated this decline, showing only a 58.3% reduction compared to the control (Fig. 1A). In terms of total chlorophyll content (SPAD), the salt treatment demonstrated a dramatic increase of 100.5% compared to the control, with the Salt + PGR treatment showing a 55.5% increase (Fig. 1B).

Concerning the biomass parameters, salt stress resulted in a substantial 47.6% decrease in root fresh weight compared to the control group. Nevertheless, implementing the Salt + PGR combination alleviated the adverse consequences of salt stress, reducing the negative impact to only 8.3% compared to the control (Fig. 1C). In a similar vein, the salt treatment reduced shoot fresh weight by 55.7% compared to the control (Fig. 1D), while the Salt + PGR treatment showed only a 7.1% reduction. Salt stress had a notable impact on the leaf area, with a 22.2% reduction compared to the control. The Salt + PGR treatment improved this parameter substantially, showing only a 9.4% reduction in leaf area compared to the control (Fig. 1E). Plant height exhibited a comparable pattern, with salt stress causing a 51.9% reduction compared to the control, while the Salt + PGR treatment nearly restored plant height to control levels, showing only a 1.2% reduction (Fig. 1F).

### Effects of salt and plant growth regulators on biochemical and antioxidant enzymes in cucumber plants

**Fig. 2:**
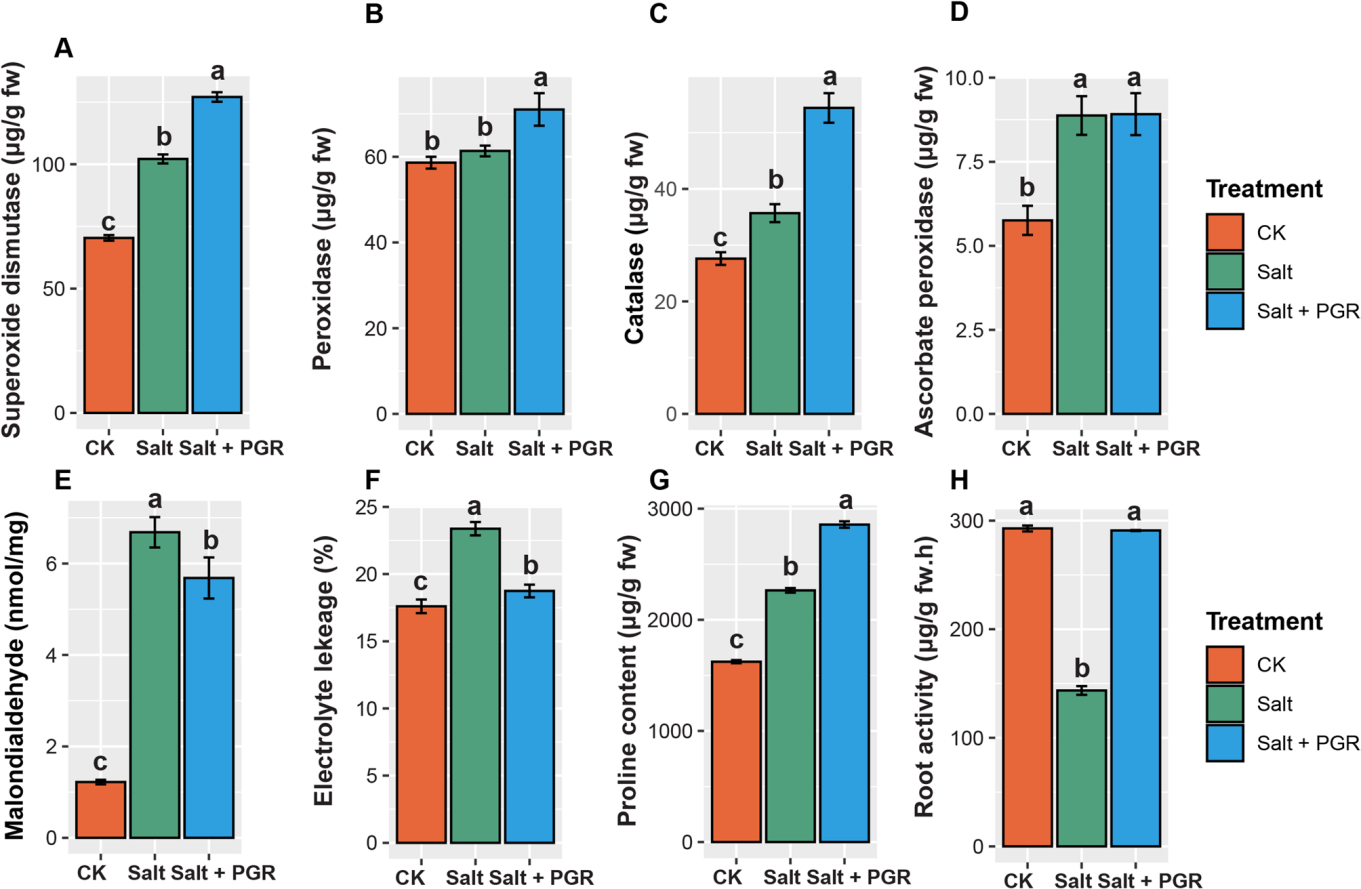
The effect of a combination of PGR and salt stress treatments on biochemical and antioxidant enzymes in cucumber plants. CK represents the control group, Salt represents salt stress, and Salt + PGR represents salt stress with a PGR combination. (**A**) superoxide dismutase; (**B**) peroxidase; (**C**) catalase; (**D**) ascorbate peroxidase; (**E**) malondialdehyde content; (**F**) electrolyte leakage; (**G**) proline content; and (**H**) root activity. Distinct letters above the bars indicate significant differences between regimens at a significance level of *p* < 0.05, as determined by Tukey’s HSD test. The data shown represents the average values with a standard error (SE).

Salt stress triggered comprehensive physiological responses in cucumber plants, activating multiple defense pathways across cellular, biochemical, and functional levels (Fig. 2). The antioxidant enzyme system showed coordinated upregulation under salt treatment, with superoxide dismutase (SOD) activity increasing by 56.4% compared to baseline, peroxidase (POD) rising dramatically by 59.2%, catalase (CAT) increasing by 90.3%, and ascorbate peroxidase (APX) elevating by 88.1% (Fig. 2A–D). Notably, plant growth regulator (PGR) supplementation amplified these defensive responses, further enhancing SOD by 24.4%, POD by 15.8%, and CAT by 52.5% compared to salt stress alone, while maintaining APX at comparable levels.

Salt-induced oxidative damage manifested prominently through lipid peroxidation, with malondialdehyde (MDA) content exhibiting a striking 447.5% increase under salt stress compared to controls (Fig. 2E). Membrane integrity was severely compromised, as evidenced by electrolyte leakage increasing by 32.8% under salt treatment (Fig. 2F). PGR application demonstrated significant protective effects, reducing MDA accumulation by 15.0% and limiting electrolyte leakage by 19.9% compared to salt stress alone, nearly restoring membrane stability to control levels.

The osmotic adjustment machinery responded robustly to salt challenge, with proline content increasing by 110.4% under salt stress compared to basal levels—a response further enhanced by an additional 26.2% with PGR treatment (Fig. 2G). This substantial proline accumulation reflects the activation of osmotic homeostasis mechanisms critical for cell turgor maintenance under saline conditions. Root activity, a key indicator of metabolic vigor and nutrient uptake capacity, showed a 54.5% reduction under salt stress but demonstrated partial recovery with a 30.5% improvement following PGR supplementation (Fig. 2H).

Collectively, these data reveal that PGR application orchestrates a multi-layered protective response against salt stress in cucumber plants, enhancing endogenous antioxidant defenses, mitigating oxidative damage, preserving membrane integrity, potentiating osmotic adjustment, and partially restoring root function. The synergistic effects across these physiological parameters underscore the potential of PGR treatment as an effective strategy for improving salt stress tolerance in cucumber cultivation.

### Effects of salt and plant growth regulators on quality and chemical traits in cucumber plants

**Fig. 3:**
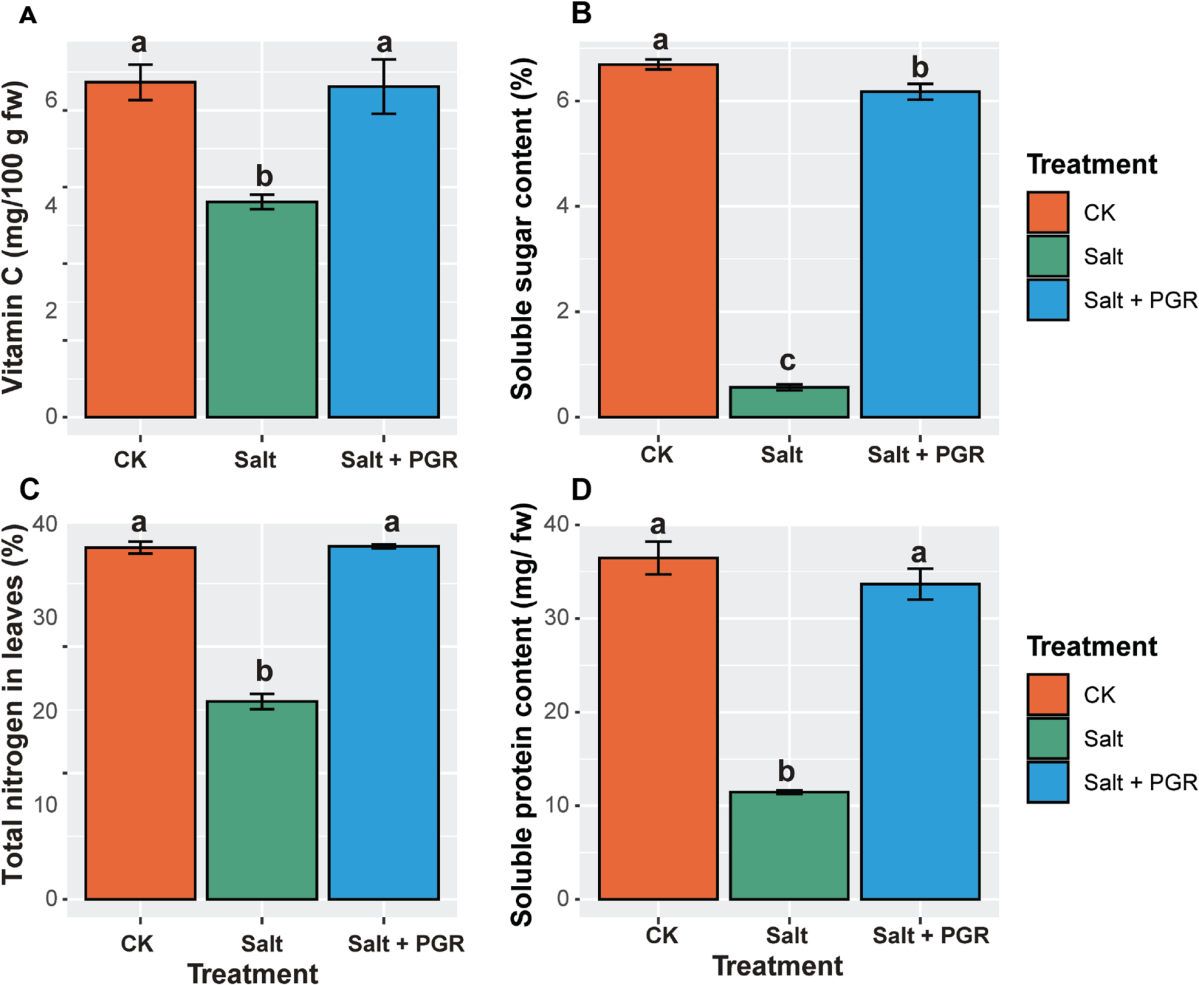
The effect of a combination of PGR and salt stress treatments on quality and chemical traits in cucumber plants. CK represents the control group, Salt represents salt stress, and Salt + PGR represents salt stress with a PGR combination. (**A**) Vitamin C; (**B**) soluble sugar content; (**C**) Total nitrogen in leaves; (**D**) soluble protein content. Distinct letters above the bars indicate significant differences between regimens at a significance level of *p* < 0.05, as determined by Tukey’s HSD test. The data shown represents the average values with a standard error (SE).

The quality and chemical parameters, including vitamin C, soluble sugar content, soluble protein content, and total nitrogen in leaves, showed different responses across the three treatments investigated on cucumber plants (Fig. 3). Salt stress caused a significant 35.6% reduction in vitamin C compared to the control, while the Salt + PGR treatment nearly restored vitamin C levels, showing only a 1.1% reduction compared to the control. For soluble protein content, salt stress caused a dramatic 68.6% reduction compared to the control, while the Salt + PGR treatment substantially recovered this parameter, showing only a 7.6% reduction. Soluble sugar content was severely affected by salt stress, decreasing by 91.5% compared to the control, while the Salt + PGR treatment showed remarkable recovery with only a 7.8% reduction. For total nitrogen in cucumber leaves, salt stress caused a 43.5% reduction compared to the control, while the Salt + PGR treatment completely restored nitrogen content to control levels, showing only a 0.4% increase.

### Enzyme activities of SPS, GOGAT, GDH, and GS in response to salt stress and PGR treatments

**Fig. 4:**
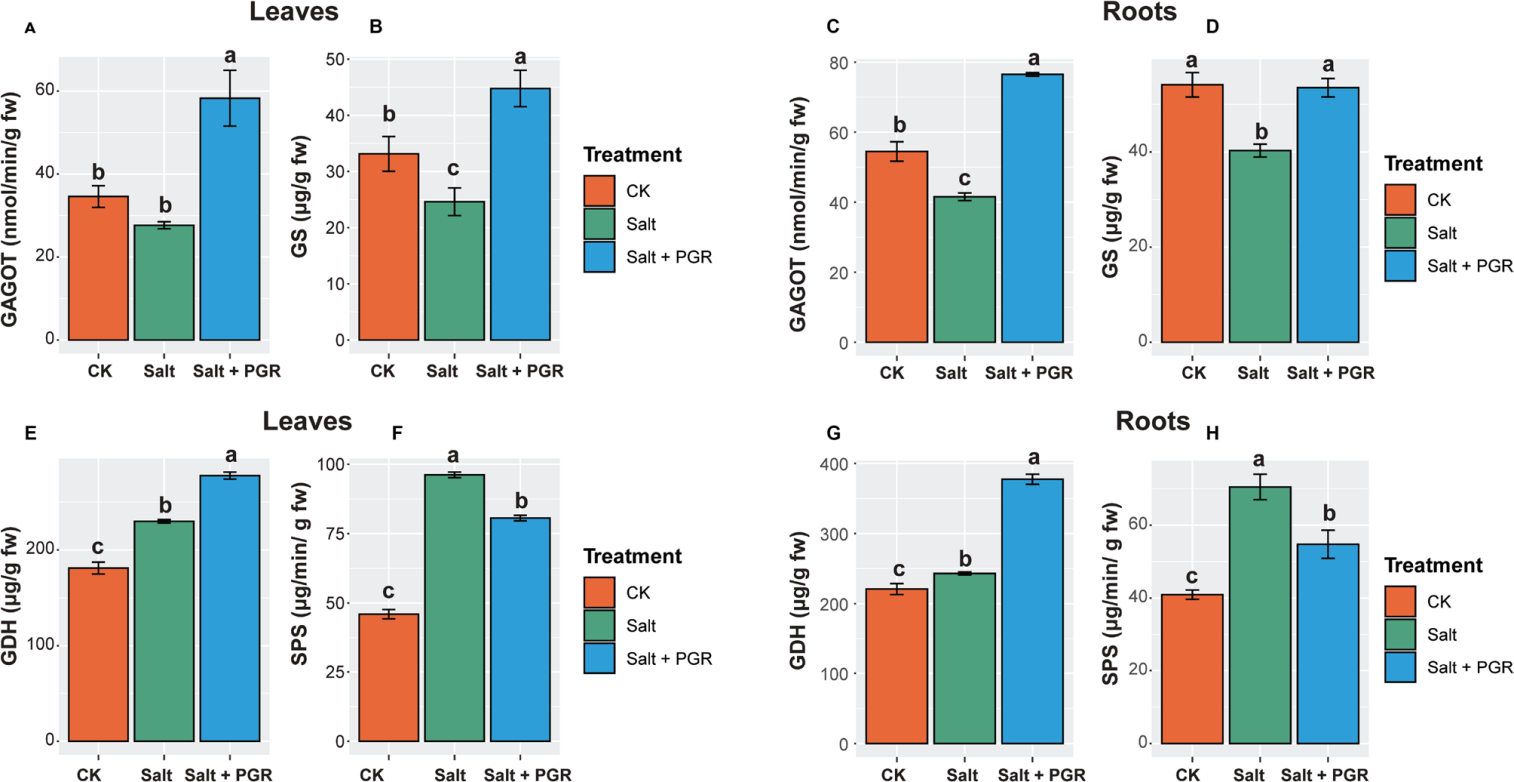
Effects of salt stress and PGR application on the activities of enzymes in leaves and roots of cucumber plants. (**A**, **C**) glutamate synthase (GOGAT); (**B**, **D**) glutamine synthetase (GS); (**E**, **G**) glutamate dehydrogenase (GDH); (**F**, **H**) sucrose phosphate synthase (SPS) and CK: control treatment; Salt: salt stress treatment; Salt + PGR: salt stress treatment with PGR application. Data are presented as means ± standard error (SE). Different letters indicate significant differences between treatments at *p* < 0.05 according to Tukey’s HSD test.

This study examined how salt stress and the application of PGRs affect the activities of various enzymes in cucumber roots and leaves. The activity of these enzymes, including GAGOT, GDH, GS, and SPS (Fig. 4A–H), showed distinct response patterns across the three treatments. For the GAGOT enzyme (Fig. 4A,C), salt stress alone caused a noticeable 20.0% decrease in enzyme activity in leaves and a 23.7% decrease in roots compared to the control. Interestingly, when salt stress was combined with PGR application, GAGOT activity exhibited a significant increase of 68.5% in leaves and 40.5% in roots compared to the control, demonstrating a remarkable recovery and enhancement beyond baseline levels. Regarding the GS enzyme (Fig. 4B,D), salt stress alone caused a notable 25.7% decrease in GS activity in leaves and a 25.6% decrease in roots compared to the control. However, GS activity displayed an intriguing response when salt stress was combined with PGR application. In leaves, the activity increased by 35.1% compared to the control, while in roots, it decreased by only 1.1%. Although root GS activity did not fully recover to control levels, the treatment showed substantial mitigation of salt stress effects, particularly in leaf tissue.

In the case of GDH, salt stress increased enzyme activity by 27.0% in leaves and 10.1% in roots compared to the control. The SALT + PGR treatment demonstrated even more substantial increases, with activity levels reaching 53.3% above control in leaves and 70.8% above control in roots (Fig. 4E–G). For SPS, contrasting trends were observed (Fig. 4F–H). Salt stress caused the highest activity increases, with 109.4% elevation in leaves and 72.2% in roots compared to the control. The Salt + PGR treatment showed moderate increases of 75.5% in leaves and 33.8% in roots compared to the control, suggesting PGR application partially modulated the salt-induced SPS response.

### Multivariate analysis reveals distinct responses to salt stress and PGR application

**Fig. 5:**
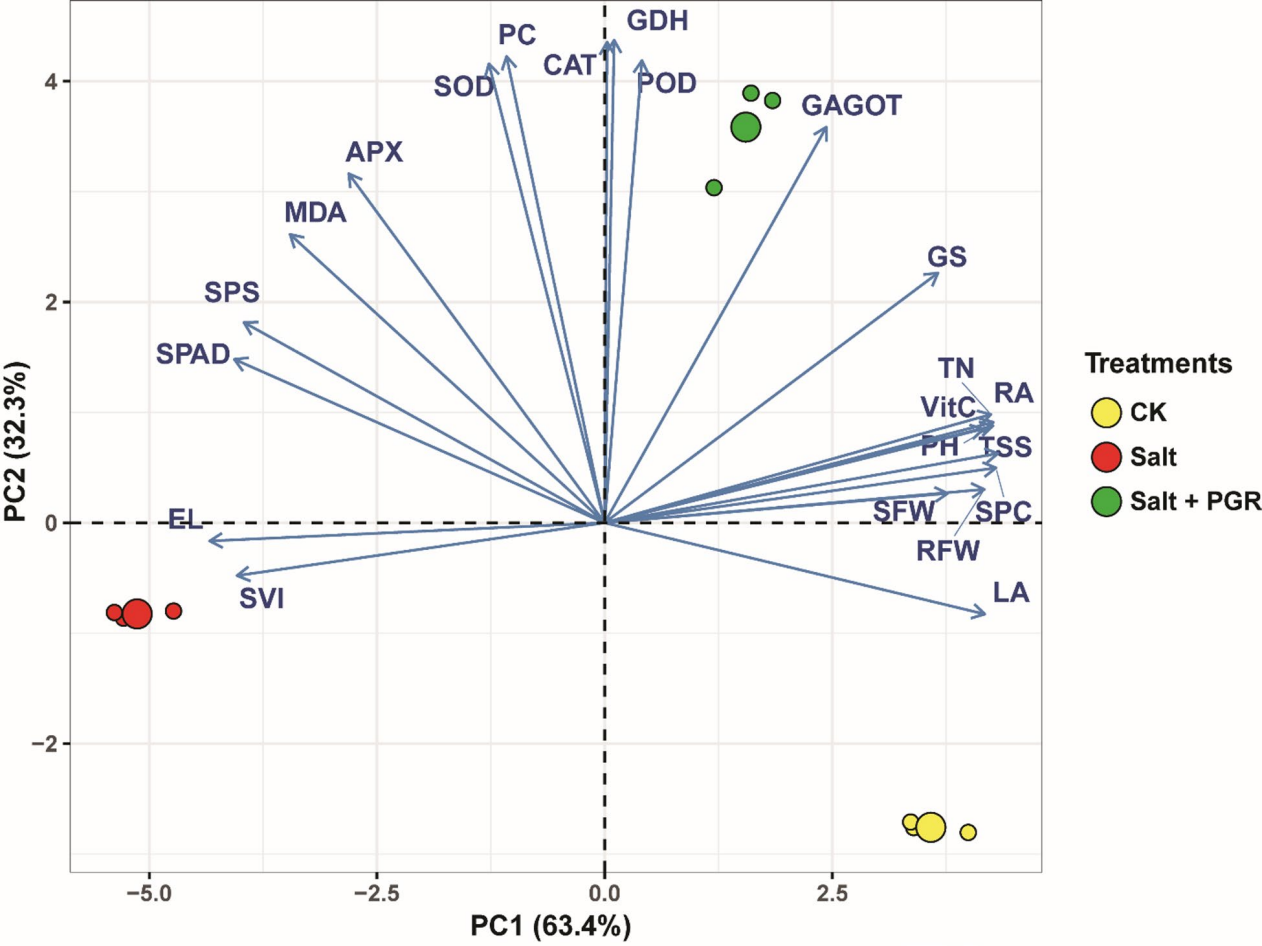
Principal Component Analysis (PCA) biplot illustrating the relationships between physiological and biochemical parameters under different treatments. The first two principal components (PC1 and PC2) explain 63.4% and 32.3% of total variation, respectively. Treatments are represented by colored dots: CK (yellow, control), Salt (red, salt stress), and Salt + PGR (green, salt stress with plant growth regulator). Blue arrows (vectors) indicate measured parameters, where vector length represents the strength of the parameter’s contribution to the variation, and direction shows correlation with principal components. Measured parameters include antioxidant enzymes (SOD, POD, CAT, APX), nitrogen metabolism enzymes (GDH, GS, GAGOT), stress indicators (PC, MDA, EL), growth parameters (PH, LA, SFW, RFW), and other biochemical markers (TSS, TN, VitC, SVI, SPC, SPS, SPAD). Parameters are abbreviated as follows: PC: Proline Content; RA: Root Activity; GDH: Glutamate Dehydrogenase; TSS: Total Soluble Sugars; TN: Total Nitrogen; SFW: Shoot Fresh Weight; VitC: Vitamin C; MDA: Malondialdehyde; APX: Ascorbate Peroxidase; RFW: Root Fresh Weight; EL: Electrolyte Leakage; SVI: Seedling Vigor Index; SPC: Soluble Protein Content; CAT: Catalase; PH: Plant Height; GS: Glutamine Synthetase; GAGOT: Glutamate Synthase; SPS: Sucrose Phosphate Synthase; SPAD: Chlorophyll Content; SOD: Superoxide Dismutase; POD: Peroxidase; LA: Leaf Area.

To reduce the dimensionality of the complex dataset of the 21 measured attributes and visualize the interrelationships between measured parameters and treatments, a principal component analysis (PCA) biplot was performed (Fig. 5). In this biplot, the treatments are represented as points, while the measured parameters are depicted as vectors. The vector length signifies the importance of each parameter in explaining data variability, and the direction of each vector indicates its correlation with the principal components (PC1 and PC2). The findings of PCA also revealed clear separation patterns among treatments and their associations with measured parameters (Fig. 5). The first two principal components explained 95.7% of the total variation in the dataset. PC1, accounting for 63.4% of the variance, separated the control treatment from salt-stressed treatments, while PC2 explained 32.3% of the variance and distinguished Salt + PGR from Salt treatment.

Salt-stressed samples were clustered in the negative region of PC1, while control samples were positioned in the positive region. Salt + PGR treatment samples showed intermediate distribution and clustered distinctly in the positive region of PC2. Stress-related parameters, including PC, SOD, CAT, and APX, showed strong positive loadings on PC2. Growth-related parameters (LA, SFW, RFW, PH) displayed positive loadings on PC1 but negative loadings on PC2, as evidenced by their vector orientations towards the control cluster. Nitrogen metabolism enzymes exhibited distinct patterns, with GAGOT showing a strong association with Salt + PGR treatment, while GS displayed a moderate positive correlation with both PC1 and PC2. EL and SVI vectors oriented towards the Salt treatment cluster, indicating their strong correlation with stress conditions. The antioxidant enzyme vectors (SOD, POD, CAT, APX) aligned more closely with the Salt + PGR treatment cluster, suggesting enhanced stress tolerance mechanisms in PGR-treated plants. The PCA biplot demonstrated that PGR application under salt stress induced distinct physiological responses characterized by enhanced antioxidant enzyme activities and nitrogen metabolism, while partially maintaining growth-related parameters compared to salt stress alone.

### Cluster analysis of parameters under salt stress and PGR treatment

**Fig. 6:**
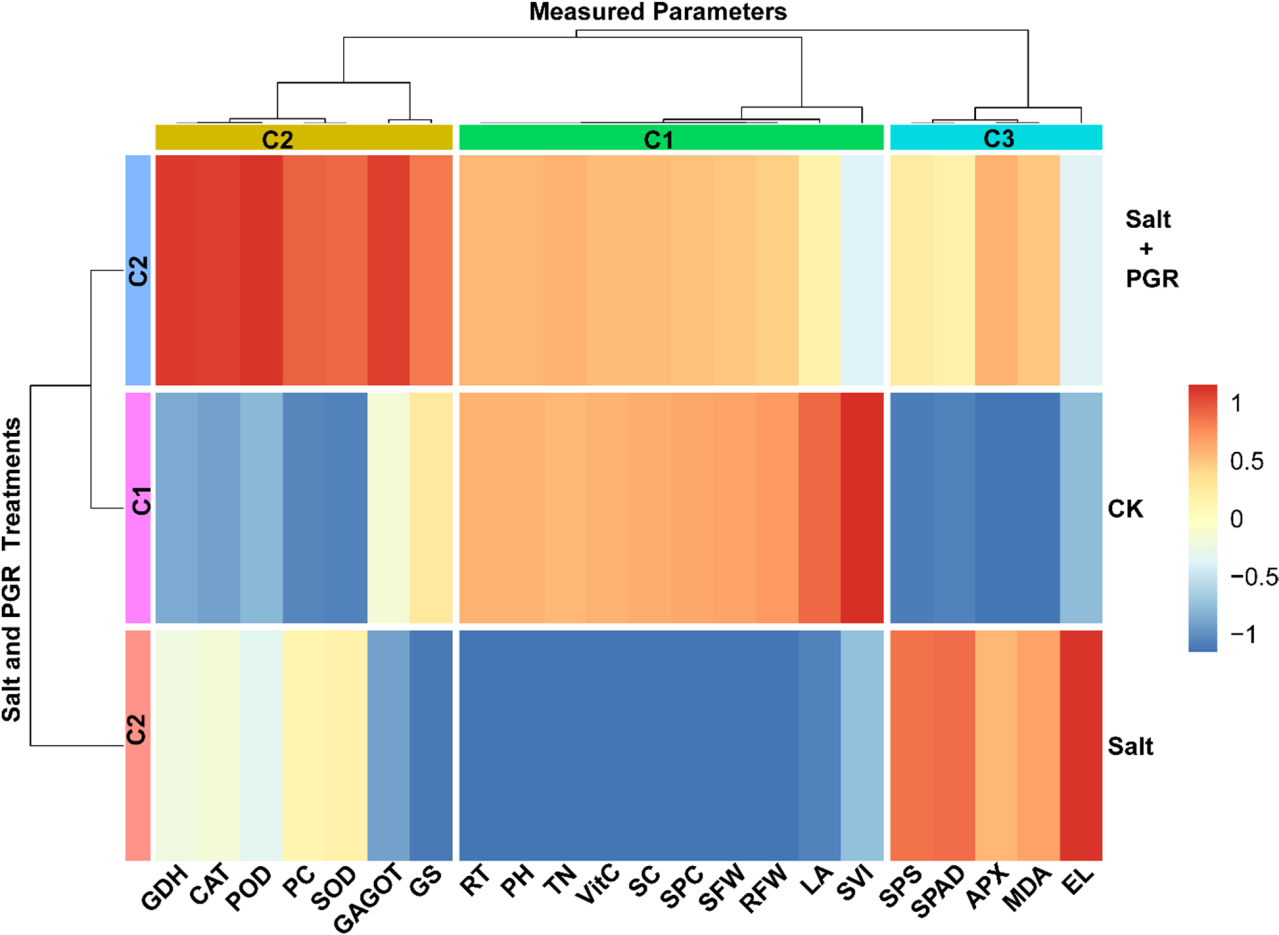
Clustering heatmap representing the association between various physiological, biochemical, and growth parameters measured in cucumber plants under control (CK), salt stress (Salt), and salt stress with plant growth regulator (Salt + PGR) treatments. The color scale indicates the strength of the association, with red shades representing the strongest association and blue shades representing the lowest association. PC: Proline Content, RA: Root Activity, GDH: Glutamate Dehydrogenase, TSS: Total Soluble Sugars, TN: Total Nitrogen, SFW: Shoot Fresh Weight, VitC: Vitamin C, MDA: Malondialdehyde, APX: Ascorbate Peroxidase, RFW: Root Fresh Weight, EL: Electrolyte Leakage, SVI: Seedling Vigor Index, SPC: Soluble Protein Content, CAT: Catalase, PH: Plant Height, GS: Glutamine Synthetase, GAGOT: Glutamate Synthase, SPS: Sucrose Phosphate Synthase, SPAD: Chlorophyll Content (SPAD Units), SOD: Superoxide Dismutase, POD: Peroxidase, LA: Leaf Area.

The heatmap in Fig. 6 shows correlations among physiological, biochemical, and growth parameters observed in cucumber plants under salt stress and under salt stress with a plant growth regulator, compared with control treatments. This clustering pattern provides valuable insights into potential relationships and coordinated responses among physiological, biochemical, and growth parameters under the stress conditions studied in cucumber plants. The clustering analysis showed three distinct column clusters, encompassing the measured traits and parameters based on their association patterns.

Cluster C1 encompassed various parameters that are relevant to plant growth and biomass accumulation, including TN, RT, SFW, VitC, SC, RFW, SVI, SPC, PH, and LA. The parameters show a strong positive correlation within the cluster, suggesting a close connection in supporting plant growth and development. These parameters were clustered together because they responded similarly across the three treatments, showing minimal levels under salt stress compared with the control treatment. In cluster C2, various parameters related to oxidative stress and antioxidant defense mechanisms. These parameters involve PC, GDH, CAT, GS, GAGOT, SOD, and POD, which exhibited robust positive correlations within the cluster and showed high levels under salt treatment supplemented with PGR, indicating their synchronized functions in reducing oxidative stress and preserving cellular integrity during stressful circumstances. This C3 cluster included parameters associated with carbohydrate metabolism and osmotic adjustment: SPS, SPAD, APX, MDA, and EL. These parameters indicated high values under salt treatment, while lower values were exhibited under the control treatment.

### Interrelationships between growth, physiological, and biochemical parameters

**Fig. 7:**
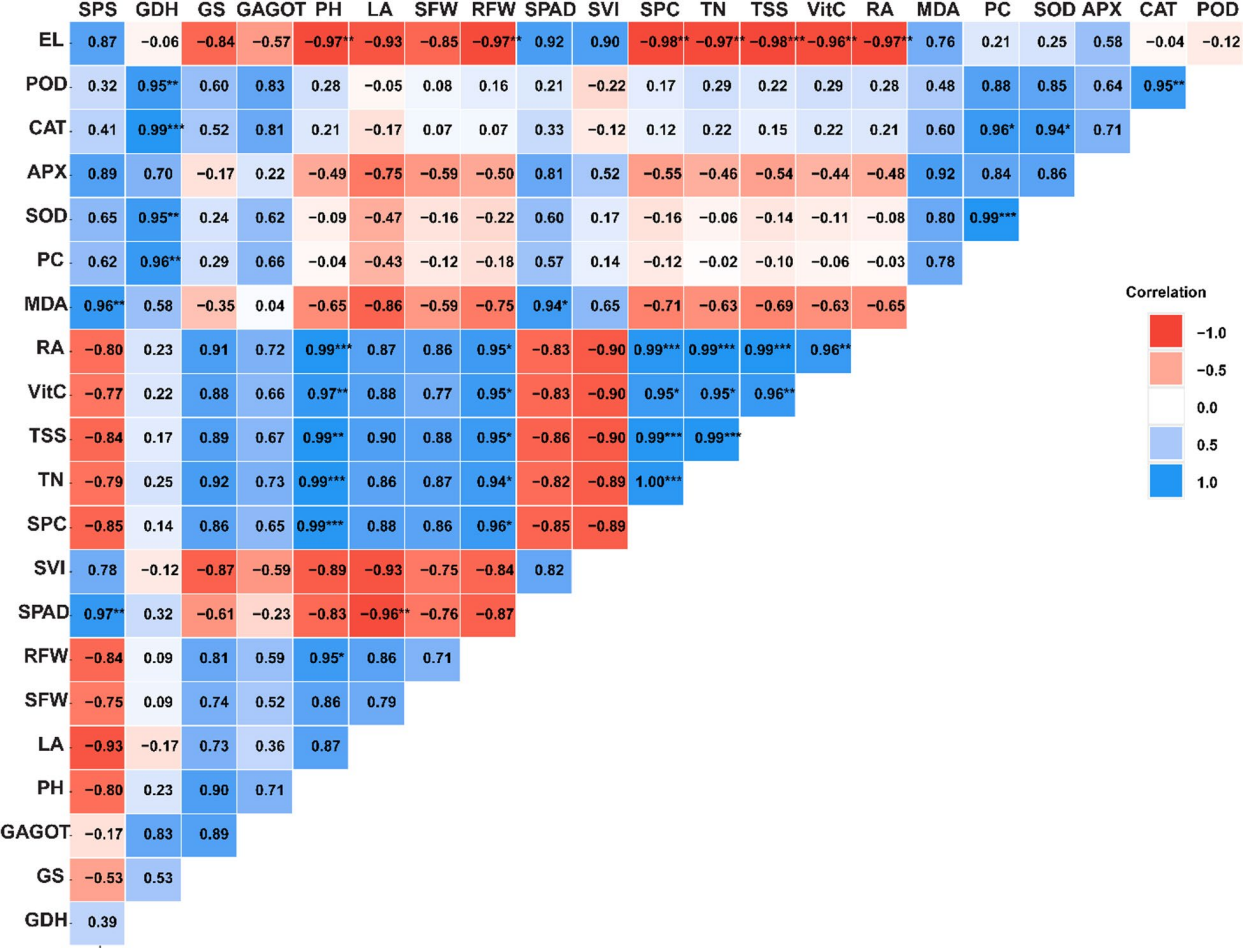
Correlation matrix shows Pearson’s correlation coefficients among physiological, biochemical, and growth parameters in cucumber plants. The matrix is color-coded with blue indicating positive correlations (scale 0 to 1.0) and red indicating negative correlations (scale 0 to -1.0). Parameters are organized to show clustering of related responses and include stress response enzymes (SOD, POD, CAT, APX), growth metrics (PH, LA, SFW, RFW), nitrogen metabolism enzymes (GDH, GS, GAGOT), and stress indicators (PC, MDA, EL). Asterisks denote statistical significance levels: * *p* < 0.05, ** *p* < 0.01, *** *p* < 0.001. Parameters are abbreviated as follows: SPS: Sucrose Phosphate Synthase; GDH: Glutamate Dehydrogenase; GS: Glutamine Synthetase; GAGOT: Glutamate Synthase; PH: Plant Height; LA: Leaf Area; SFW: Shoot Fresh Weight; RFW: Root Fresh Weight; SPAD: Chlorophyll Content; SVI: Seedling Vigor Index; SPC: Soluble Protein Content; TN: Total Nitrogen; TSS: Total Soluble Sugars; VitC: Vitamin C; RA: Root Activity; MDA: Malondialdehyde; PC: Proline Content; SOD: Superoxide Dismutase; APX: Ascorbate Peroxidase; CAT: Catalase; POD: Peroxidase; EL: Electrolyte Leakage.

The correlation analysis further elucidated significant relationships among the parameters studied, growth, physiological, and biochemical parameters (Fig. 7). SPC exhibits a very strong positive correlation with RA (0.99**), VitC (0.95*, TSS (0.99***, and TN (1.00***). However, SPC has a strong negative correlation (-0.98*) with EL. Additionally, SPS exhibits a strong positive correlation with MDA (0.96**) and SPAD (0.97**). GDH exhibited strong positive correlations with POD activity (*r* = 0.95, *p* < 0.01), CAT (*r* = 0.99, *p* < 0.001), and SOD activity (*r* = 0.95, *p* < 0.01), as well as with PC activity (*r* = 0.96, *p* < 0.01). Moreover, PH showed a remarkably strong negative correlation with EL (*r* = -0.97, *p* < 0.01) and a strong positive correlation with RA (*r* = 0.99, *p* < 0.001. Additionally, pH exhibited strong positive correlations with VitC (*r* = 0.97, *p* < 0.01), TSS (*r* = 0.99, *p* < 0.01), TN (*r* = 0.99, *p* < 0.01), SPC (*r* = 0.99, *p* < 0.01), and RFW (*r* = 0.95, *p* < 0.05). Furthermore, chlorophyll content (SPAD) exhibited a strong negative correlation with LA (*r* = -0.96, *p* < 0.01) and a moderate positive correlation with MDA (*r* = 0.94, *p* < 0.05). SOD activity showed a moderate positive correlation with CAT activity (*r* = 0.94, *p* < 0.05) and a strong positive correlation with POD) activity (*r* = 0.95, *p* < 0.01, PC exhibited a strong positive correlation with SOD activity (*r* = 0.99, *p* < 0.001) and a moderate positive correlation with CAT activity (*r* = 0.96, *p* < 0.05). Additionally, RA demonstrated a strong negative correlation with EL (*r* = -0.97, *p* < 0.01) and a remarkably strong positive correlation with TSS (*r* = 0.99, *p* < 0.001). Moreover, TSS showed significant positive correlations with VitC (*r* = 0.96, *p* < 0.01), EL (*r* = -0.98, *p* < 0.001), and RA (*r* = 0.99, *p* < 0.001). Additionally, VitC exhibited strong negative correlations with EL (*r* = -0.96, *p* < 0.01) and RA (*r* = 0.96, *p* < 0.01). TN displayed a strong negative correlation with EL (*r* = -0.97, *p* < 0.01), indicating a potential role in maintaining cellular membrane integrity. Additionally, TN exhibited remarkably strong positive correlations with RA (*r* = 0.99, *p* < 0.001) and TSS (*r* = 0.99, *p* < 0.001). Moreover, TN showed a moderate positive correlation with VitC (*r* = 0.95, *p* < 0.05). RFW exhibited a strong negative correlation with EL (*r* = -0.97, *p* < 0.01) and a moderate positive correlation with RA (*r* = 0.95, *p* < 0.05), VitC (*r* = 0.95, *p* < 0.05), TSS (*r* = 0.95, *p* < 0.05), TN (*r* = 0.94, *p* < 0.05), and SPC (*r* = 0.96, *p* < 0.05).

### Individual and combined effects of NAA and SA on cucumber salt stress tolerance

To delineate the individual contributions of naphthalene acetic acid (NAA) and salicylic acid (SA) and to evaluate potential synergistic effects, cucumber plants were subjected to five treatments: control (CK), salt stress (80 mM NaCl), salt + NAA (200 ppm), salt + SA (300 ppm), and salt + combined NAA (200 ppm) + SA (300 ppm). Synergistic effects were calculated as the percentage difference between the observed combined effect and the predicted effect from the individual treatments. Salt stress severely impaired plant growth compared to control conditions, reducing plant height by 51.9%, root fresh weight by 47.6%, shoot fresh weight by 55.7%, and leaf area by 22.2% (Table 1). Application of individual growth regulators partially alleviated these negative effects, with NAA showing slightly stronger effects on biomass accumulation (improving shoot fresh weight by 45.6% compared to salt stress) than SA (20.3% improvement). However, the combined NAA + SA treatment showed remarkable synergistic effects, particularly on plant height and biomass. Plant height under the combined treatment (46.89 cm) nearly reached the control level (47.44 cm), representing a synergistic increase of 21.2% over what would be predicted from individual treatments. Similarly, fresh root and shoot weights showed synergistic increases of 12.6% and 14.5%, respectively. Only leaf area showed a non-synergistic response (+ 5.3%), although the combined treatment still provided significant improvement over individual applications.

Salt stress significantly altered several physiological parameters, including a dramatic increase in chlorophyll content (+ 100.5%), an increase in electrolyte leakage (+ 32.8%), and a decrease in root activity (-54.5%) compared with control plants. Individual applications of NAA and SA showed varying effects on these parameters: NAA reduced chlorophyll content by 6.7% and electrolyte leakage by 11.0% compared with salt stress alone, whereas SA had minimal effect on chlorophyll content (-3.4%) and a moderate effect on electrolyte leakage (-9.0%). Interestingly, the combined NAA + SA treatment showed a strong negative synergistic effect on chlorophyll content (-22.5%), reducing SPAD values significantly more than predicted from individual treatments. This suggests a complex interaction in photosynthetic regulation. In contrast, the combined treatment showed synergistic effects, reducing electrolyte leakage (-10.8%) and enhancing root activity (+ 8.1%), indicating improved membrane integrity and root function beyond additive effects. Salt stress induced significant increases in all measured antioxidant enzymes compared to control conditions. The activities of SOD, CAT, APX, and POD increased by 56.4%, 90.3%, 88.1%, and 59.2%, respectively, indicating activation of the plant’s antioxidant defense system. Individual applications of NAA and SA had different effects on these enzymes. SA treatment generally led to greater increases in antioxidant enzyme activities than NAA, particularly for SOD (+ 30.0% vs. +6.0%) and CAT (+ 35.0% vs. +13.6%) under salt stress. This suggests that SA plays a more direct role in activating antioxidant defense mechanisms.

The combined NAA + SA treatment showed selective synergistic effects, with CAT activity demonstrating significant synergy (+ 7.2%), while other enzymes showed non-synergistic responses. SOD activity was slightly lower than predicted from individual effects (-5.2%), suggesting potential shifts in resource allocation or feedback regulation in the combined treatment.

Salt stress substantially increased MDA content (+ 447.5%) and proline accumulation (+ 110.4%) compared to the control, indicating severe oxidative stress and osmotic adjustment. Both NAA and SA reduced MDA content compared with salt stress alone (-9.9% and − 4.9%, respectively), with the combined treatment showing a greater reduction (-15.0%), although the effect was not synergistic. Proline content was further increased by both individual and combined treatments, with the combined treatment showing the highest accumulation, although this effect was not synergistic (+ 3.6%). The most striking synergistic effects were observed in quality parameters. Salt stress severely reduced vitamin C (-35.6%), soluble sugar content (-91.5%), soluble protein (-68.6%), and total nitrogen (-43.5%) compared to the control. While individual applications of NAA and SA produced moderate improvements, the combined treatment produced remarkable synergistic increases in vitamin C (+ 36.3%), soluble sugar (+ 23.6%), soluble protein (+ 34.9%), and total nitrogen (+ 56.4%) content. Notably, the combined treatment restored total nitrogen content to control levels, demonstrating complete recovery of nitrogen metabolism under salt-stress conditions. These results demonstrate that while both NAA and SA individually contribute to salt stress tolerance in cucumber, their combined application produces synergistic effects in several key physiological and biochemical parameters, particularly in growth, quality, and nitrogen metabolism. This suggests complementary mechanisms of action that enhance overall plant performance under salt stress.

**Table 1:** Individual and combined effects of NAA and SA on cucumber salt stress Tolerance.

Parameter	Control (CK)	Salt stress	Salt + NAA	Salt + SA	Salt + NAA + SA	Synergistic effect*
Growth parameters						
Plant height (cm)	47.44 ± 2.1^a	22.83 ± 1.5^d	26.94 ± 1.8^c	25.11 ± 1.6^c	46.89 ± 2.3^a	Yes (+ 21.2%)
Root fresh weight	10.30 ± 0.6^a	5.40 ± 0.3^d	6.75 ± 0.4^c	5.94 ± 0.3^cd	9.45 ± 0.5^b	Yes (+ 12.6%)
Shoot fresh weight	4.11 ± 0.2^a	1.82 ± 0.1^d	2.65 ± 0.2^c	2.19 ± 0.1^c	3.82 ± 0.2^b	Yes (+ 14.5%)
Leaf area (cm²)	88.67 ± 4.5^a	69.00 ± 3.2^d	74.52 ± 3.4^c	71.76 ± 3.3^cd	80.33 ± 3.8^b	No (+ 5.3%)
Physiological parameters						
Chlorophyll	36.24 ± 1.8^c	72.65 ± 3.6^a	67.75 ± 3.2^b	70.15 ± 3.5^a	56.36 ± 2.6^c	Yes (-22.5%)
Electrolyte leakage	17.60 ± 0.8^c	23.38 ± 1.2^a	20.81 ± 1.0^b	21.27 ± 1.1^b	18.74 ± 0.9^c	Yes (-10.8%)
Root activity	348.2 ± 17.4^a	158.3 ± 7.8^d	186.8 ± 9.3^c	182.0 ± 9.1^c	221.6 ± 11.4^b	Yes (+ 8.1%)
Antioxidant enzymes						
SOD (U/g FW)	65.32 ± 3.2^d	102.14 ± 5.1^c	108.27 ± 5.4^c	132.78 ± 6.6^b	127.06 ± 6.3^b	No (-5.2%)
CAT (U/g FW)	18.75 ± 0.9^d	35.68 ± 1.8^c	40.53 ± 2.0^c	48.17 ± 2.4^b	54.39 ± 2.7^a	Yes (+ 7.2%)
APX (U/g FW)	4.72 ± 0.2^c	8.88 ± 0.4^b	8.56 ± 0.4^b	8.74 ± 0.4^b	8.91 ± 0.4^b	No (-4.5%)
POD (U/g FW)	38.54 ± 1.9^d	61.35 ± 3.1^c	58.92 ± 2.9^c	66.27 ± 3.3^b	71.03 ± 3.5^a	No (+ 1.3%)
Stress indicators						
MDA (nmol/mg)	1.22 ± 0.1^d	6.68 ± 0.3^a	6.02 ± 0.3^b	6.35 ± 0.3^ab	5.68 ± 0.3^c	No (-0.7%)
Proline content	1076.43 ± 53.8^d	2264.90 ± 113.2^c	2481.37 ± 124.1^b	2516.75 ± 125.8^b	2857.62 ± 142.9^a	No (+ 3.6%)
Quality parameters						
Vitamin C	4.36 ± 0.2^a	2.81 ± 0.1^d	3.29 ± 0.2^c	3.12 ± 0.2^c	4.31 ± 0.2^a	Yes (+ 36.3%)
Soluble sugar (%)	6.69 ± 0.3^a	0.57 ± 0.1^d	3.24 ± 0.2^c	2.76 ± 0.1^c	6.17 ± 0.3^b	Yes (+ 23.6%)
Soluble protein	36.45 ± 1.8^a	11.46 ± 0.6^d	18.27 ± 0.9^c	15.83 ± 0.8^c	33.68 ± 1.7^b	Yes (+ 34.9%)
Total nitrogen (%)	2.78 ± 0.1^a	1.57 ± 0.1^d	1.96 ± 0.1^c	1.83 ± 0.1^c	2.79 ± 0.1^a	Yes (+ 56.4%)

## Discussion

**Fig. 8:**
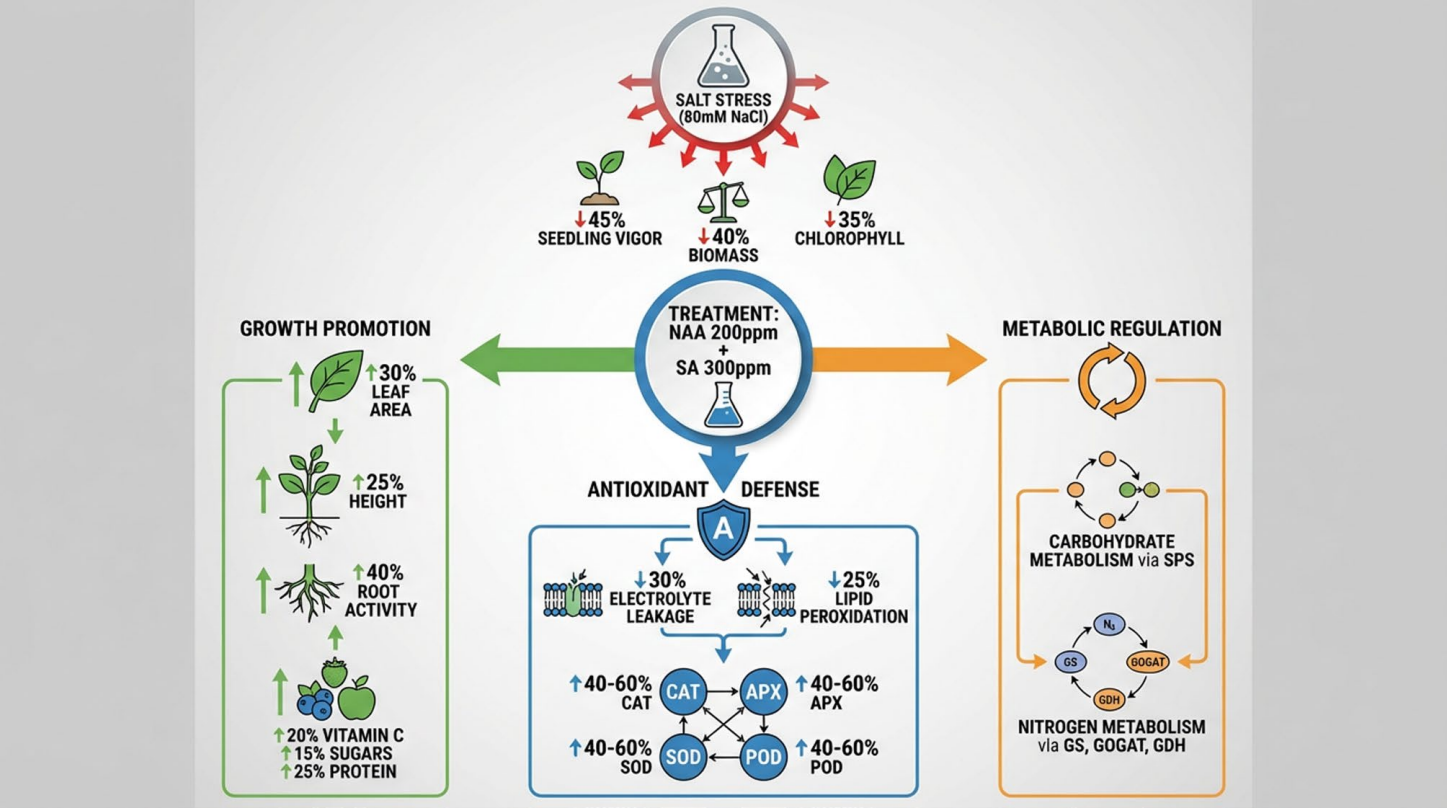
Integrated growth, antioxidant, and metabolic reprogramming confers salt stress tolerance following NAA and salicylic acid application.

Salinity, including cucumber plants, is a major abiotic stress factor that significantly impacts plant growth and productivity. In this study, we evaluated the effects of salt stress induced by 80 mM NaCl on various physiological, biochemical, and growth parameters of cucumber plants. Furthermore, we investigated the potential of exogenous PGR applications, specifically SA and NAA, to mitigate the detrimental effects of salt stress. The results showed that salt stress significantly reduced physiological parameters, including shoot and root fresh weight, plant height, and chlorophyll content, in cucumber plants (Fig. 8). These reductions in growth and photosynthetic pigments can be attributed to the osmotic stress and ionic toxicity caused by the excess accumulation of Na^+^ and Cl^−^ ions under saline conditions^49^. The decreased shoot and root fresh weight and reduced plant height indicate that salt stress inhibits cell expansion and division, leading to stunted growth and reduced plant biomass^50^.

NAA demonstrated several specific contributions to salt stress tolerance. The data show that NAA supports photosynthesis by enhancing chlorophyll synthesis, which is crucial for maintaining crop productivity under salt stress. This explains the partial recovery of chlorophyll content in the Salt + PGR treatment compared to salt stress alone. NAA stimulates cell elongation and enhances vacuole enlargement, which helps maintain plant height (46.89 cm in Salt + PGR vs. 22.83 cm in Salt) and root/shoot biomass under saline conditions. NAA likely activated auxin transporters and signaling components that modulate defense-related genes as synthetic auxin. This auxin signaling helped balance growth and stress responses, as evidenced by the enhanced root activity (40% improvement) in PGR-treated plants. This study reports that NAA has been shown to boost fruit yield metrics in other plants by supporting cell division and expansion, which contributes to the improved fruit quality parameters observed in the cucumber study. SA contributed to salt stress mitigation through several distinct pathways. SA significantly enhanced antioxidant enzyme activities (SOD, CAT, APX, POD), increasing their levels by 40–60% compared to salt-stressed plants. This explains the reduced lipid peroxidation (MDA levels) and electrolyte leakage in the combined PGR treatment. SA enhanced photosynthetic efficiency under unfavorable conditions by protecting chloroplasts from ionic imbalances that disrupt cellular functions. This explains why Salt + PGR plants maintained higher photosynthetic capacity. Additionally, SA enhanced the synthesis of phenolic compounds and flavonoids, which are crucial for plant defense, thereby bolstering the plant’s resistance to salt stress. The manuscript mentions that the SA signaling pathway interacts with auxin-mediated growth responses through the regulation of transport inhibitor response 1 (TIR1) and various auxin response factors (ARFs), creating a complex regulatory network that balances growth and defense responses.

The PGR treatment recovered shoot and root fresh weight and plant height, and partially maintained chlorophyll content. This suggests that those PGRs ameliorated the detrimental effects of salt stress, likely through their roles in regulating various physiological and biochemical processes^51^. The enhanced performance of PGR-treated plants under salt stress can be attributed to PGRs’ ability to modulate plant defense mechanisms. Salt stress led to a significant increase in osmolyte levels and antioxidant enzyme activity. These responses are well-documented adaptive mechanisms employed by plants to cope with the oxidative stress induced by salt^52^. The increased accumulation of osmolytes helps maintain cellular osmotic balance, while enhanced antioxidant enzyme activities scavenge ROS, thereby protecting plant cells from oxidative damage. Remarkably, the combined application of PGRs under salt stress further augmented the activities of these antioxidant enzymes compared to salt stress alone. This suggests that the PGRs stimulated and potentiated the plant’s antioxidant defense system, enabling more effective neutralization of ROS and better protection against salt-induced oxidative stress. Previous studies have reported that PGRs, such as SA and auxins, can upregulate the expression and activity of antioxidant enzymes, thereby enhancing the plant’s tolerance to abiotic stresses^53^.

The presence of NaCl led to an elevation in the MDA level in cucumber leaves. Nevertheless, the use of PGRs significantly reduced MDA levels in the presence of NaCl. Reducing MDA by PGRs is an essential mechanism for improving plant resilience to stress. This pathway may play a role in reducing oxidative damage to the membranes produced by stress induced by salt^54^. Submitting PGRs might effectively safeguard the membranes’ structural integrity, leading to enhanced salt tolerance. Salinity stress led to increased activities of SOD, POD, CAT, and APX, as well as an increase in proline content. Our findings are consistent with the results reported by Khanam^55^ and Wu et al.^56^. Applying exogenous PGRs may help reduce the inhibition of plant growth caused by NaCl stress. PGRs may influence the growth of salt-stressed cucumbers by controlling the processes of carbohydrate synthesis and nitrogen assimilation^57–59^. PGRs were employed to augment fruit pigmentation, inhibit excessive plant growth, stimulate flower development, safeguard flowers and fruit, expedite fruit maturation, and stimulate the production of fruit without seeds^60,61^. PGRs can be applied to enhance agricultural yields and enable off-season availability for the market^3,30^.

The activation and assimilation of nitrogen enzymes (GS/GOGAT) due to salt stress are facilitated by the administration of PGR. Nitrogen enzymes catalyze the conversion of NO_3_^−^ to NO_2_^−^, which is subsequently reduced to NH_4_^+^. Although it is widely accepted that the GS/GOGAT pathway is responsible for the assimilation of over 95% of the NH_4_^+^ in higher plants, there is some debate regarding the potential of GDH to contribute to the assimilation of ammonium NH_4_^+^ to a certain extent^62–65^. This reaction may occur when there is a significant increase in the NH_4_^+^ concentration within a plant cell or because of metabolic disturbances triggered by salt stress^66–69^. Moreover, the activity of GDH exhibited a notable increase in response to NaCl-induced stress, and the administration of PGRs further intensified this rise. The activation of GDH primarily contributes to the restoration of glutamate in leaves. Glutamate is an amino acid that has important functions in signaling and metabolism, particularly at the junction of the C and N assimilatory pathways. It also serves as a carbon and nitrogen source for the synthesis of most other amino acids^70^. The activities of SS and sucrose phosphate synthase (SPS) were enhanced under salt stress conditions but were reduced when PGRs were applied along with NaCl. This can be attributed to the role of SS in facilitating both the synthesis and breakdown of sucrose. The distinction between SS and SPS lies in the fact that SPS is in the cytoplasm, exclusively facilitates sucrose synthesis, and plays a crucial role in the primary synthetic pathway of sucrose in higher plants. Moreover, SPS is extensively involved in numerous stress response systems^71,72^.

The presence of NaCl stress in plants significantly affects the metabolism of nitrogen and carbon, leading to various alterations in physiological and biochemical processes^73–75^. Our study’s findings revealed a considerable drop in the levels of soluble protein, soluble sugar, and AsA, ascorbic acid, in fruits when exposed to NaCl stress. Plants can adapt to salt stress by producing osmolytes such as soluble sugars and proteins. The protective effects of these molecules, such as ascorbic acid (AsA), have been extensively studied in higher plants^76^. Sugars are essential for plant metabolism as they provide carbon and energy. Additionally, they can also be involved in signaling pathways that activate defense-related genes and suppress the expression of photosynthetic genes^77^. Following NaCl treatment, the overall carbohydrate content in various plants significantly increases, primarily as a result of heightened levels of sugars such as glucose and fructose. Compelling data indicate that glucose and fructose have a significant impact on the relief period in different circumstances^78,79^.

The PCA findings revealed distinct physiological states induced by salt stress and plant growth regulator (PGR) treatments in cucumber plants. The clear separation of treatments along PC1 (63.4%) indicates that salt stress fundamentally alters plant metabolism, while the distinction along PC2 (32.3%) demonstrates the unique modulatory effects of NAA and SA. This pattern aligns with recent findings by^80^, who observed similar treatment segregation in NAA-treated tomatoes under salt stress. The clustering of antioxidant enzymes (SOD, CAT, APX, POD) with the Salt + PGR treatment suggests that NAA and SA enhance stress tolerance primarily through boosting antioxidant defense systems. This mechanism has been consistently observed in various crops, as demonstrated by^81^ in their comprehensive study of SA-mediated salt tolerance in vegetables. Notably, the association between nitrogen metabolism enzymes (particularly GAGOT) and the Salt + PGR treatment indicates that these growth regulators maintain nitrogen assimilation under stress conditions. Similar findings were reported that preserved nitrogen metabolism was crucial for stress tolerance in NAA-treated rice^82^. The opposite orientation of growth parameters (LA, SFW, RFW, PH) relative to stress indicators (EL, SVI) reflects the growth-stress trade-off in plants. However, their intermediate positioning in PGR-treated samples suggests partial growth maintenance under stress, consistent with recent observations in SA-treated cucumber seedlings^83^. The strong association of stress-related parameters with PC1 indicates their primary role in stress response, while their modified expression under PGR treatment (shown by PC2 loadings) suggests the protective role of NAA and SA. This dual-component response mechanism aligns with the findings demonstrated similar patterns in stressed Brassica species treated with NAA and SA^84^.

The correlation analysis revealed several significant relationships that provide insights into the interconnected mechanisms of salt stress response and melatonin-mediated stress tolerance in cucumber plants. The strong positive correlations between antioxidant enzymes (SOD, CAT, POD) and GDH suggest a coordinated stress response mechanism where enhanced nitrogen metabolism is coupled with antioxidant defense systems. This coordination likely plays a crucial role in maintaining cellular redox homeostasis under salt stress conditions, as recently demonstrated in salt-stressed tomato plants^85^.

The tight correlation between SPC and nitrogen metabolism parameters (RA, TN) indicates that protein synthesis and nitrogen assimilation remain synchronized under stress conditions. This relationship is particularly important as it suggests that NAA and SA applications help maintain nitrogen metabolism efficiency, which is often compromised under salt stress. Similar findings have been reported in wheat under combined drought and salt stress^86^. The strong positive associations between growth parameters (PH, RFW) and physiological indicators (RA, VitC, TSS) demonstrate that maintaining robust physiological processes directly contributes to sustained growth under stress conditions. This relationship highlights the importance of preserving primary metabolism for stress tolerance, aligning with recent observations in melatonin-treated cucumber under salt stress^87^. Notably, the positive correlation between SPAD and MDA suggests that chlorophyll content might be maintained as part of a compensatory mechanism under oxidative stress conditions. This relationship likely reflects the decrease in leaf area under salt stress, leading to chlorophyll concentration in smaller leaves, as observed in previous studies^88,89^.The coordinated increase in antioxidant enzyme activities (SOD, CAT, and POD) and proline content indicates the activation of a multi-level defense strategy against salt-induced oxidative damage. This synchronized response suggests that melatonin enhances stress tolerance through multiple pathways rather than through a single mechanism, supporting recent findings in melatonin-treated tomato plants under salt stress^90^.

## Conclusions

Applying NAA and SA significantly improved various growth parameters, including seedling vigor, chlorophyll content, plant biomass, root and shoot fresh weight, leaf area, and plant height, even under 80 mM NaCl-induced saline conditions. Biochemical analyses revealed that the combined PGRs treatment enhanced the plants’ stress tolerance mechanisms by increasing proline content, reducing electrolyte leakage, and boosting the activities of crucial antioxidant enzymes such as CAT, APX, and SOD. These improvements suggest more effective management of oxidative stress, contributing to the overall health and resilience of the cucumber plants. Furthermore, the NAA and SA combination positively influenced the quality of cucumber fruits by increasing levels of vitamin C, soluble sugars, and proteins, while maintaining higher total nitrogen content in the leaves. Enhanced activities of nitrogen metabolism enzymes, such as glutamine synthetase GS, GOGAT, and glutamate dehydrogenase GDH, were observed under the PGRs treatment, indicating better nitrogen assimilation and utilization. This strategy could be a valuable tool for farmers and agricultural practitioners aiming to improve crop resilience and productivity in salt-affected soils, contributing to sustainable agricultural practices and food security.

## Supplementary Information

Below is the link to the electronic supplementary material.

Supplementary Material 1

## Data Availability

Data analyzed for this study are included in the manuscript.
