# Supplementary figures and images for: Synergistic effect of naphthalene acetic acid and salicylic acid on the growth and tolerance mechanism of cucumber under salt stress
Source: Sci Rep. 2026 Mar 14;16:9203. doi: 10.1038/s41598-026-39439-x (PMC12996297; doi:10.1038/s41598-026-39439-x)

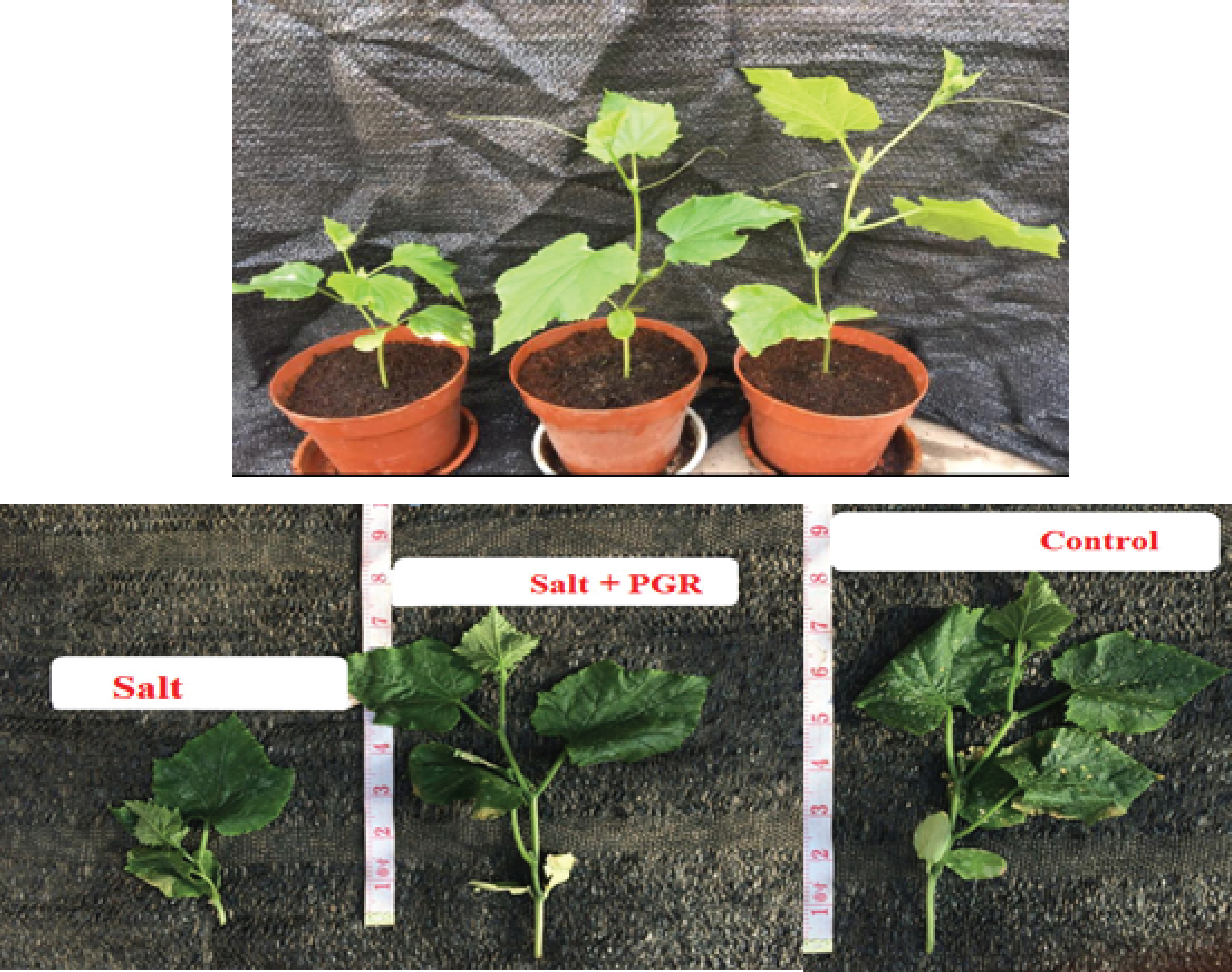

Supplement: Supplementary file 1 — Supplementary Material 1 [file 41598_2026_39439_MOESM1_ESM.png]
